# Large reversible caloric effect in FeRh thin films via a dual-stimulus multicaloric cycle

**DOI:** 10.1038/ncomms11614

**Published:** 2016-05-19

**Authors:** Yang Liu, Lee C. Phillips, Richard Mattana, Manuel Bibes, Agnès Barthélémy, Brahim Dkhil

**Affiliations:** 1 Laboratoire Structures, Propriétés et Modélisation des Solides, CentraleSupélec, CNRS-UMR8580, Université Paris-Saclay, Grande Voie des Vignes, Châtenay-Malabry Cedex 92295, France; 2 Unité Mixte de Physique, CNRS, Thales, University Paris Sud, Université Paris-Saclay, Palaiseau 91767, France

## Abstract

Giant magnetocaloric materials are promising for solid-state refrigeration, as an alternative to hazardous gases used in conventional cooling devices. A giant magnetocaloric effect was discovered near room temperature in near-equiatomic FeRh alloys some years before the benchmark study in Gd_5_Si_2_Ge_2_ that launched the field. However, FeRh has attracted significantly less interest in cooling applications mainly due to irreversibility in magnetocaloric cycles associated with the large hysteresis of its first-order metamagnetic phase transition. Here we overcome the irreversibility via a dual-stimulus magnetic-electric refrigeration cycle in FeRh thin films via coupling to a ferroelectric BaTiO_3_ substrate. This experimental realization of a multicaloric cycle yields larger reversible caloric effects than either stimulus alone. While magnetic hysteretic losses appear to be reduced by 96% in dual-stimulus loops, we show that the losses are simply transferred into an elastic cycle, contrary to common belief. Nevertheless, we show that these losses do not necessarily prohibit integration of FeRh in practical refrigeration systems. Our demonstration of a multicaloric refrigeration cycle suggests numerous designs for efficient solid-state cooling applications.

## Abstract (web summary)

Refrigeration devices based on giant magnetocaloric materials are hampered by an irreversible caloric effect associated with large magnetic hysteresis. Here, Liu *et al*. report a multicaloric refrigeration cycle in FeRh thin films coupled to a ferroelectric BaTiO_3_ substrate, demonstrating reversibility and conversion of hysteretic losses.

## Graphical abstract

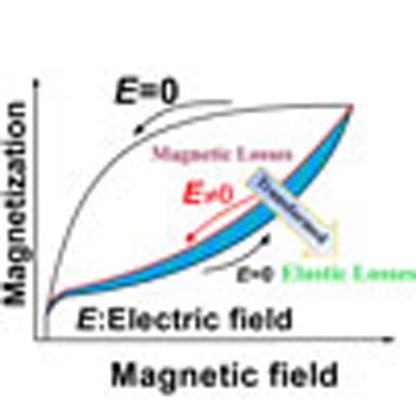

Caloric materials, which undergo an adiabatic temperature change Δ*T* or isothermal entropy change Δ*S* when some external stimulus is applied or withdrawn, are highly sought after for new refrigeration solutions to replace current vapor-cycle cooling technologies^1^. The most prominent calorics are ferroically ordered materials that often exhibit giant caloric effects near their ferroic transitions. Depending on the external stimulus, effects^1^ may be called magnetocaloric (magnetic field), electrocaloric (electric field), elastocaloric (uniaxial stress) or barocaloric (hydrostatic pressure), the last two being very similar and now considered together as mechanocaloric effects. In multiferroic materials and heterostructures^2^, multicaloric effects^1^,^3^ can exploit multiple sources of entropy and be driven by either single stimulus^3^ or multiple stimuli (applied/removed simultaneously or sequentially)^1^. While theoretical descriptions of multicaloric effects are now emerging^3^,^4^,^5^, experiments are still limited to probing individual effects^6^,^7^. In particular, multiple-stimulus caloric cycles have yet to be realized, and their advantage over conventional cycles is not clear.

FeRh exhibits a giant negative magnetocaloric effect (MCE) near room temperature^8^, arising from an unusual first-order transition near 350 K between antiferromagnetic (AFM) and ferromagnetic (FM) order, whose physical origin is still under debate^9^. The giant MCE was discovered in FeRh^8^ some years before the better-known result in Gd_5_Si_2_Ge_2_ compounds^10^, and its caloric parameters Δ*S*∼20 J K^−1^ kg^−1^ and Δ*T*∼13 K (in 2 T magnetic field)^8^ make FeRh a highly competitive magnetocaloric material even today^1^,^11^. As the transition is accompanied by a unit cell volume expansion of about 1%, FeRh is also barocaloric^12^,^13^ and elastocaloric^14^. However, the broad hysteresis often associated with the transition^10^,^11^,^15^ (and to a lesser extent the high cost of Rh) are key drawbacks for applications. The former results in a dramatic degradation of MCE versus refrigeration cycles as observed by recent direct measurements^13^, which is in contrast to the nominal reproducible MCE results calculated either by differential scanning calorimetry^12^ or by magnetization-magnetic field curves^16^. The irreversibility of the MCE is associated with very broad or incomplete transitions, which arise in disordered thick films^17^ and in very thin FeRh films^18^,^19^. While the irreversibility and hysteresis losses in other caloric materials with first-order magnetic transitions have been reduced by doping^20^, or introducing porosity^21^, such reductions have remained elusive in the case of FeRh.

In this study, we propose and realize a magnetic-electric multicaloric cycle in a multiferroic heterostructure, and use it to solve a real and longstanding problem, that is, a large hysteresis that impeded reversibility in an otherwise promising magnetocaloric material. Our heterostructure consists of a thin film (TF) of equiatomic FeRh alloy on a single-crystal BaTiO_3_ (BTO) substrate. We previously showed that FeRh/BTO heterostructures exhibit the largest magnetoelectric coupling coefficient^22^,^23^, implying the existence of a large effective electrocaloric effect (technically an electric field-induced mechanocaloric effect^1^, as will be discussed later) driven by a voltage applied across the BTO. Unfortunately, in our previous work^22^ the electric field-induced transition was mostly irreversible. Here we achieve a large reversible MCE in FeRh/BTO structures via a dual-stimulus multicaloric cycle that employs both magnetic and electric fields. Although dual-stimulus cycles do not remove intrinsic losses as previously claimed^24^, we show that those losses are not important enough to exclude practical applications, and we thus suggest a route to revive the potential of FeRh for magnetic cooling applications.

## Results

### Reversible MCE through multicaloric refrigeration cycle

**Figure 1:**
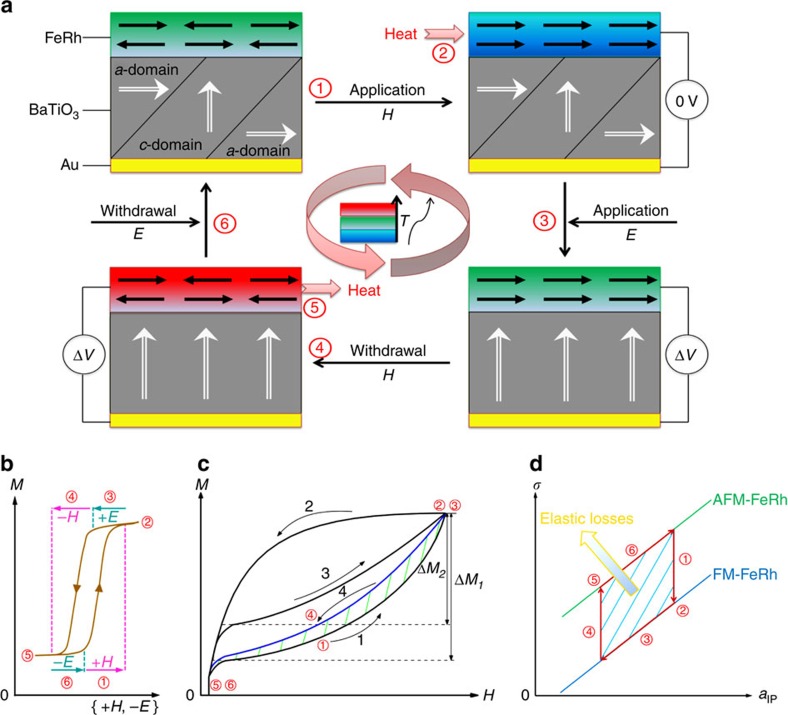
Multicaloric refrigeration cycle. (**a**) Schematic of a dual-stimulus multicaloric refrigeration cycle. (**b**) Schematic of the effect of electric and magnetic fields (*E*, *H*) on the magnetization *M* of FeRh. The electric field overcomes the hysteresis, but does not itself cause *M* to change. (**c**) Schematic of magnetic cycles, in the space of magnetization *M* versus magnetic field *H*. The blue curve denotes a path followed in the presence of the electric field. Black uncircled numbers refer to measurement paths described in the main text. (**d**) Schematic of elastic losses, in the space of biaxial thin film stress *σ* versus average in-plane lattice constant of FeRh *a*_IP_. In **a**–**d**, the red circled numbers 1–6 correspond to the steps described in the main text, and in **b**–**d** arrows indicate the sequence of proposed measurements.

Our dual-stimulus multicaloric refrigeration cycle is schematized in Fig. 1a. While a similar multicaloric cycle using pressure was discussed elsewhere^24^, the full cycle was not demonstrated experimentally. For the following proposal only, we suppose that all steps can be performed fast enough to avoid heat leakage from the FeRh TF into the BTO substrate, aided, for example, by a thermal barrier layer that preserves strain coupling. Under these circumstances, we imagine the rapid changes to be adiabatic in principle.

Initially, the FeRh TF is in its AFM phase and the BTO substrate has a mixed 90° a/c ferroelastic domain structure. The cycle begins (step 1) by applying (increasing) a magnetic field *H* adiabatically, favouring the FM phase of FeRh and causing the FeRh film to cool down. This is followed by absorbing heat from an external source keeping *H* constant (step 2), due to the negative MCE^1^,^8^,^11^,^12^,^13^,^15^,^16^. At step 3, the electric field *E* is applied adiabatically to the BTO substrate resulting in more *c*-domains and thus favouring the AFM phase of FeRh TFs^22^,^23^. The magnetic field *H* is then removed (decreased) adiabatically causing the FeRh to heat up (step 4), followed by a heat ejection towards the external source (step 5). Finally, the electric field *E* is removed (step 6). Our proposed refrigeration cycle differs from purely magnetic ones^11^ by the introduction of electric field in steps 3 and 6, whose effect on FeRh is in principle similar to that of a magnetic field (Fig. 1b), but which in practice does not stimulate the FeRh transition. The typical MCE in FeRh follows a path with a large virgin effect, followed by a smaller reversible trajectory^13^,^16^ (path 1—2—3, Fig. 1c). By selecting appropriate values of electric field *E*, we may apparently cancel the hysteresis and achieve repeatable anhysteretic magnetic loops (path 1—4, Fig. 1c), although the electric field-induced elastic strain applied in steps 3 and 6 converts an amount of mechanical work into heat that is similar to the magnetic work saved (Fig. 1d). Instead, we achieve reversible changes that are considerably larger than those achievable in a single-stimulus cycle with either magnetic or electric field. We now present an experimental demonstration of a slow, isothermal analogue of the proposed cycle in FeRh/BTO.

We grew a ∼40-nm-thick epitaxial FeRh film at 630 °C by magnetron sputtering from an equiatomic Fe_50_Rh_50_ target onto a 500-μm-thick single-crystal (001) BTO substrate. The high film quality, including epitaxy and Fe/Rh order parameter, were confirmed by reflection high-energy electron diffraction images and X-ray diffraction scans, respectively^22^. A 50-nm-thick Au layer was sputtered on the back side of the BTO as a bottom electrode, and voltages were applied across the thickness of the BTO substrate. Details of our sample preparation can be found elsewhere^22^,^23^. We use a film around twice as thick as in our previous work^22^,^23^ to avoid the particularly large hysteretic losses of very thin FeRh films^18^,^19^,^22^,^23^, which are due to grain size reduction^18^ and substrate-induced strain^22^. Magnetization measurements were carried out in a Quantum Design superconducting quantum interference device (SQUID) magnetometer with a maximum accessible temperature of 400 K and the capability to apply magnetic field up to 5 T. Given that most experimental works on the caloric effect were characterized by isothermal entropy changes rather than direct measurement of adiabatic temperature changes^1^,^11^, we use magnetization as a proxy for the progress of the first-order transition, because the fast heat exchange between TFs and substrates prevents us from carrying out direct thermal measurements^25^.

**Figure 2:**
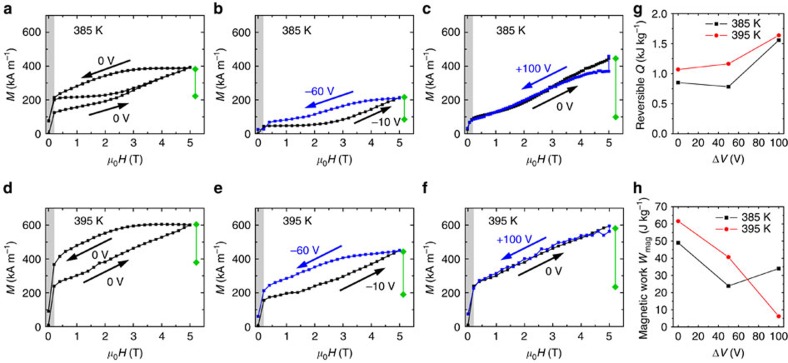
Isothermal magnetic measurements. (**a**–**f**) Experimental magnetization versus magnetic field curves during isothermal (**a**,**d**) magnetic, (**b**–**c**,**e**–**f**) magnetic-electric cycles at (**a**–**c**) 385 K and (**d**–**f**) 395 K. In (**a**), the remagnetization curve after a full cycle is also shown. The grey shaded region (*μ*_0_*H*<0.25 T) is dominated by rearrangement of ferromagnetic domains. Diamond-headed green markers indicate the reversible magnetization change Δ*M* achieved in each cycle. (**g**) Reversible isothermal heat *Q* and (**h**) magnetic work done, versus the change in voltage Δ*V* at *μ*_0_*H*=5 T.

Figure 2 shows isothermal magnetization (*M*) versus magnetic field (*H*) curves measured on increasing and decreasing magnetic field at 385 K (Fig. 2a–c) and 395 K (Fig. 2d–f) immediately after heating from room temperature, either with no applied electric field (Fig. 2a,d), or magnetized and demagnetized under various moderate voltages Δ*V* close to the coercive voltage (± ∼50 V) of the BTO^22^,^23^: −10 V and −60 V (Fig. 2b,e), and 0 V and 100 V (Fig. 2c,f), where the voltage values chosen to manipulate the fraction of BTO *a*- and *c*-domains, as explained previously^23^. We chose two temperatures within the broad FeRh transition to show that the shapes of the curves have no systematic temperature dependence, although absolute *M* values are higher at 395 K where FM-FeRh is generally favoured. Similarly, in Fig. 2b,c the BTO contained mostly *c*-domains throughout, resulting in generally lower *M* values. Our calculations consider only the region *μ*_0_*H*>0.25 T where the FM phase is magnetically saturated, and thus the magnetization represents the amount of FM phase present. We define the reversible range of FeRh magnetization Δ*M* as the range in *M* that is covered in both increasing and decreasing *H* sweeps (green bars, Fig. 2a–f). The irreversibility is apparent in Fig. 2a where we have included the results of a second magnetic field sweep, during which the magnetization remains rigidly within this reversible range.

Our main result is that optimized multicaloric cycles (Fig. 2c,f) yield larger reversible Δ*M*, and hence the reversible isothermal heat *Q*=|*T*Δ*S*| pumped during one magnetocaloric cycle (Fig. 2g) is also larger compared with either magnetic field alone (Fig. 2a,d) or electric field alone (Fig. 3 of ref. 22). Here we use *Q*=*TρV*Δ*S*_max_(Δ*M*/Δ*M*_max_), where the FeRh film has mass density *ρ*=9,760 kg cm^−3^, *V* is the film volume and we use literature values Δ*M*_max_=1,190 e.m.u. per cm^3^ (ref. 12) and Δ*S*_max_=17 J K^−1^ kg^−1^ (ref. 9) for the complete transition because the SQUID maximum temperature of 400 K prevents us from acquiring the full data set to estimate Δ*S* via the Maxwell relation^26^.

Interestingly, we also reduce the magnetic work (or magnetic hysteresis losses) *W*_mag_, defined by the difference in area ∫*M* d*H* between curves with increasing and decreasing *H*, by up to 96% (Fig. 2h) because the voltage-induced strain effect arising from the BTO domain structure^22^,^23^ brings the FeRh close to the onset of transformation before each variation of *H*. Such a reduction would be superior to previous work on hysteresis reduction in Ni-Mn-In-Co/Pb(Mg_1/3_Nb_2/3_)O_3_-PbTiO_3_(PMN-PT) (ref. 27), but as we introduce work in the elastic cycle (Fig. 1b) we do not make this claim, and we use the term hysteresis losses to describe *W*_mag_ here. Comparing our work with ref. 27, the epoxy bonding in Ni-Mn-In-Co/PMN-PT is much weaker than our epitaxial contact, and the resulting relatively weak magnetoelectric coupling may limit the reversibility of MCE for applications. The important mechanical work was also overlooked in the elastic cycle^27^. Therefore we emphasize the need for caution when analysing multicaloric refrigeration cycles of the type we propose in Fig. 1.

To understand whether losses in our FeRh/BTO system would prohibit practical applications, in Table 1, we compare them with undoped and doped Gd_5_Si_2_Ge_2_ (ref. 20) in terms of the fundamental parameters of magnetocaloric cooling devices^1^, that is, the isothermal heat *Q* and the pumped heat/total work ratio *Q*/*W*, which constitutes an upper bound to the thermodynamic coefficient of performance (COP), COP_max_, for refrigerators. Here we take *Q* for FeRh/BTO at 395 K and Δ*V*=100 V, but we use *W*=*W*_mag_ for the cycle at 395 K without voltage, because the total *W* should be approximately the same for all multicaloric cycles. While the value COP_max_=26.9 for our highly lossy FeRh TF is lower than for even undoped Gd_5_Si_2_Ge_2_, this should not at all be the dominant inefficiency in the system. For example, in a non-isothermal refrigeration cycle with a hot reservoir at *T*_h_=395 K and a temperature span (*T*_h_−*T*_c_)=8.5 K (ref. 15), the COP is bounded by the Carnot limit COP_Carnot_=*T*_c_/(*T*_h_−*T*_c_)=45.5; assuming COP_Carnot_=*Q*/*W*_Carnot_, our additional work *W* would lead to COP=*Q*/(*W*+*W*_Carnot_)=16.8 or 37% of the Carnot efficiency. These figures are competitive, and it is likely that the real COP would be dominated by other losses in the device, which in our case would include losses in the substrate ferroelectric cycle (which can themselves be reduced by optimization). We do not calculate the refrigerant capacity (ref. 20) here, as our data does not reveal the full temperature dependence of the entropy change.

Although here we report data only at 385 K and 395 K, an advantage of using FeRh TFs is that the large reversible caloric effects can be potentially achieved at any temperature within the ∼100 K wide tetragonal phase field of BTO (including room temperature) because the FeRh transition temperature can be tuned over hundreds of kelvins and through room temperature by doping with other metallic elements^28^. This extreme tunability is in contrast with, for example, the La_0.7_Ca_0.3_MnO_3_/BTO system (∼6 K) where reversible MCE is restricted to the vicinity of the rhombohedral-orthorhombic transition of BTO at ∼200 K^25^. We note that the tuning of the metamagnetic phase transition temperature (>20 K) by electric field as we have already achieved in refs 22, 23 is also ideal to achieve a large temperature span in an active magnetic regenerator^27^. Moreover, the tunability of our FeRh/BTO system is considerably greater than that in Ni-Mn-In-Co/PMN-PT (<10 K, ref. 27).

The fact that the transition from AFM phase to FM phase at 385 K is not fully achieved under 5 T (see Fig. 2a,b), in line with previous results on ultrathin FeRh TFs^18^,^19^, suggests that even larger effects could be obtained by microstructural optimization, for example, by nanopatterning to artificially introduce porosity. Also, as the strain states in FeRh are discrete, that is, related to either an *a*- or *c*-domain of BTO, it is difficult to access a wider range of strain states in FeRh^22^,^23^. In contrast, a continuous range of electro-strains can be achieved if FeRh TFs are grown on a relaxor substrate like PMN-PT, which would also fortuitously reduce the losses in the substrate (as in ref. 27).

Our results here demonstrate that the historical irreversibility of MCE in FeRh discovered almost 25 years ago^8^ can be solved by using an electric field, although the magnetic hysteresis losses are not destroyed but simply transferred to an elastic cycle. Since research in multicaloric materials has intensified recently^1^, we believe that our work will stimulate more efforts in multiferroic systems^2^ where strong mechanical coupling between ferroic order parameters may provide more diverse choices for the design of efficient solid-state refrigeration, including purely electric field-driven systems (see below). Revisiting overlooked compounds like FeRh using hybrid heterostructures can also provide a new life for many magnetocaloric materials. Alternative geometries including multilayer structures, core-shell nanoparticles or nanocomposites (NCs)^5^,^25^ may also be of interest for achieving comparable volume fractions of magnetic and electric components.

### Electric field-induced mechanocaloric effect

Finally, an optimized device may also permit a purely electric field-driven refrigeration cycle consisting of steps 3 and 6 alone. This overlooked electric field-induced mechanocaloric effect^2^ may also open a new paradigm to potentially overcome the inevitable uneconomic investments on clumsy magnets in magnetic cooling. Unlike traditional magnetocalorics^1^,^11^, the entropy change is induced on application of an electric field on BTO substrate rather than a magnetic field directly applied on FeRh (step 1) thanks to the magnetoelectric coupling. As the elastic hysteresis losses might not be critical, the novel mechanocaloric effect subjected to the electric field-induced two-dimensional stress/strain could be a low-cost approach for developing the next generation of solid-state refrigeration.

**Figure 3:**
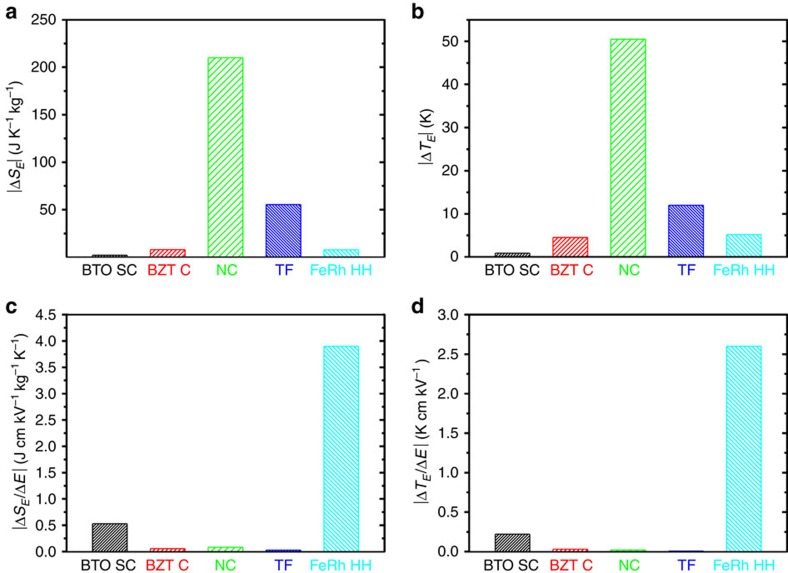
Comparison of different electric field-induced caloric effects in selected materials. Comparison of caloric effect (**a**) |Δ*S*_*E*_| and (**b**) |Δ*T*_*E*_| and strength (**c**) |Δ*S*_*E*_/Δ*E*| and (**d**) |Δ*T*_*E*_/Δ*E*| between FeRh/BaTiO_3_ hybrid heterostructure (HH) and typical giant electrocaloric materials in the literature: BaTiO_3_ single crystal (SC) (ref. 29), BaZr_0.2_Ti_0.8_O_3_ (BZT) ceramic (C) (ref. 30), P(VDF-TrFE-CFE)/Boron nitride nanosheets/Ba_0.67_Sr_0.33_TiO_3_ nanocomposite (NC) (ref. 31) and P(VDF-TrFE) thin film (TF) (ref. 32), respectively.

Figure 3a,b shows the promise of FeRh/BTO in the electric field-induced mechanocaloric effect by comparing its performance with a representative sample of competitive electrocaloric materials^29^,^30^,^31^. FeRh/BTO would perform comparably to all of these systems except for P(VDF-TrFE-CFE)/Boron nitride nanosheets/Ba_0.67_Sr_0.33_TiO_3_ NC (ref. 31), which apparently outperform all other systems. There are few mechanocaloric data available for FeRh, possibly due to concerns about irreversibility and the high price of Rh. Here we use elastocaloric parameters (|Δ*S*_*E*_|∼7.8 J K^−1^ kg^−1^ and |Δ*T*_*E*_|∼5.2 K, ref. 14) rather than larger barocaloric parameters (that is, |Δ*S*_*p*_|∼12.5 J K^−1^ kg^−1^, ref. 12) to deduce approximately the mechanocaloric effect. Interestingly, Fig. 3c,d shows that the caloric strengths |Δ*S*_*E*_/Δ*E*| (3.9 J cm V^−1^ K^−1^ kg^−1^) and |Δ*T*_*E*_/Δ*E*| (2.6 K cm kV^−1^) under an electric field Δ*E* of ∼2 kV cm^−1^ are at least 1 order of magnitude larger than previous best reports on electrocaloric effect in BTO single crystals^29^ (SC), and nearly 3 orders of magnitude larger than those in P(VDF-TrFE) TFs^32^. Our results also make a significant improvement on previous mutiferroic heterostructures: |Δ*S*_*E*_| is significantly larger than that (1.43 J K^−1^ kg^−1^) in La_0.7_Sr_0.3_MnO_3_/PMN-PT while |Δ*S*_*E*_/Δ*E*| is over 1 order of magnitude larger than that (0.20 J cm V^−1^ K^−1^ kg^−1^) in La_0.7_Sr_0.3_MnO_3_/PMN-PT^33^.

We emphasize that our multicaloric effects rely on strain-mediated coupling^2^ but not on any phase transition of BTO, and are thus exempt from the requirement that the magnetic and ferroelectric/piezoelectric layers should have similar phase transition temperatures^3^, as suggested in a recent theoretical study^34^. However, other parameters including the layer thicknesses and heat capacities are important in the eventual design of a cooling system, so that their values in a composite multiferroic should be taken into account to determine the optimal design parameters^34^. In addition, a purely magnetic heat-pump scheme without any second stimulus was proposed theoretically in FeRh without experimental evidence^35^ while our proposed multicaloric refrigeration cycle is realized by experimental support.

In summary, our results here demonstrate that the historical irreversibility of MCE in FeRh discovered almost 26 years ago^8^ can be solved by using an electric field. The dual-stimulus multicaloric cycle creates larger caloric effects, although the magnetic hysteresis losses are not destroyed but simply transferred to an elastic cycle. Since research in multicaloric materials has intensified recently^1^, we believe that our work will stimulate more efforts in multiferroic systems^2^ where strong mechanical coupling between ferroic order parameters may provide more diverse choices for the design of efficient solid-state refrigeration, including purely electric field-driven systems. Finally, revisiting overlooked compounds like FeRh using hybrid heterostructures can also provide a new life for many magnetocaloric materials.

### Data availability

The data that support the findings of this study are available from the corresponding author (Y.L. (liuyangphy52@gmail.com) or L.C.P. (lee.phillips@cantab.net)) on request.

## Additional information

**How to cite this article:** Liu, Y. *et al*. Large reversible caloric effect in FeRh thin films via a dual-stimulus multicaloric cycle. *Nat. Commun.* 7:11614 doi: 10.1038/ncomms11614 (2016).

## Figures and Tables

**Table 1:** Comparison of magnetocaloric properties between Gd_5_Ge_2_Si_2_ and FeRh.

Material	*μ*_0_*H* (T)	Isothermal heat*Q* (kJ kg^−1^)	Hysteresis loss*W* (J kg^−1^)	COP_max_
Gd_5_Ge_2_Si_2_	2	5.1	78.0	65.4
Gd_5_Ge_1.9_Si_2_Fe_0.1_	2	2.135	3.9	555.5
FeRh/BTO (without Δ*V*)	5	1.64	61.6	26.9

COP, coefficient of performance.

Comparison of isothermal heat *Q*=|*T*Δ*S*|, hysteresis loss *W* and coefficient of performance COP_max_=*Q/W*, between Gd_5_Ge_2_Si_2_ alloys (before/after doping with Fe in ref. 20) and FeRh thin films.
